# Resveratrol enhances the tolerance of *Malus hupehensis* to potassium deficiency stress

**DOI:** 10.3389/fpls.2024.1503463

**Published:** 2024-11-14

**Authors:** Zhijuan Sun, Tianchao Wang, Jianyu Li, Xiaodong Zheng, Hongjuan Ge, Guangli Sha, Changqing Ma, Qiang Zhao, Caihong Wang, Yike Tian

**Affiliations:** ^1^ College of Horticulture, Qingdao Agricultural University, Qingdao, China; ^2^ College of Life Science, Qingdao Agricultural University, Qingdao, China; ^3^ Fruit Tree and Tea Research Institute, Qingdao Academy of Agricultural Sciences, Qingdao, China

**Keywords:** resveratrol, K deficiency stress, *Malus hupehensis*, oxidative damage, ion balance

## Abstract

Potassium (K) deficiency in plants can lead to metabolic disorders and growth retardation. Currently, nearly 50% of the arable land in China is experiencing a K resource deficit, significantly hindering the development of the apple industry. Resveratrol (Res), a phytoalexin, has been extensively reported to enhance plant resistance against various abiotic stresses. However, there have been no reports on the response of Res to K^+^ deficiency stress in apples. This study aims to investigate the effect of exogenous Res on the K^+^ deficiency tolerance of *Malus hupehensis*. The results demonstrated that applying 50 μM of exogenous Res could enhance the tolerance of *M. hupehensis* to K^+^ deficiency stress. Compared to non-Res treatments, external application of Res alleviated leaf chlorosis and improved root growth in apple seedlings. Additionally, it increased antioxidant enzyme activity, thereby reducing the production of reactive oxygen species induced by K^+^ deficiency stress. Res also regulated proline and soluble sugar content to maintain osmotic balance. Moreover, Res helped maintain the balance of mineral element contents in apple seedlings and the normal K^+^: Na^+^ ratio by enhancing the influx of K^+^. Furthermore, exogenous Res regulated the expression of related kinase genes, promoting Ca^2+^ signal transduction in response to K^+^ deficiency stress and inducing the expression of K^+^ transport genes to enhance K^+^ absorption, thus supporting normal plant growth. In conclusion, this study provides a theoretical basis for the application of exogenous Res to alleviate K^+^ deficiency stress in apples.

## 1 Introduction

Potassium (K) is an essential nutrient and the most abundant cation in plants, playing a critical role in plant growth and metabolism (Wang et al., 2018, 2021). In addition to regulating ion balance within plant cells and facilitating the transmembrane transport of nutrients, K^+^ is involved in enzyme activation, protein synthesis, photosynthesis, osmotic pressure, stomatal movement, and stress resistance (Jin et al., 2024). Apples are among the most widely planted and productive horticultural fruit crops globally. In apple production, maintaining proper soil conditions and nutrient balance is crucial for ensuring optimal yield and quality (An et al., 2018). However, K^+^ deficiency has become increasingly severe due to prolonged over-cultivation and improper fertilizer application (Lu et al., 2022). Currently, approximately 52% of China’s arable land is experiencing a K^+^ shortage (Li et al., 2024). Prolonged K^+^ deficiency can cause leaf necrosis, reduced plant height, poor-quality fruits, and significantly decreased yield (Wei et al., 2022), seriously restricting the development of China’s apple industry.

K^+^ deficiency can induce osmotic stress, oxidative damage, and ion imbalance in plant cells (Tewari et al., 2007; Zhu et al., 2020; Sun et al., 2023). Osmotic stress reduces the number of stomata in leaf guard cells, decreases photosynthesis, and inhibits plant growth and development (Chen et al., 2019a; Zheng et al., 2021b). K^+^ deficiency disrupts oxygen metabolism in plants, leading to the generation of excessive reactive oxygen species (ROS), such as superoxide (O_2_^-^), hydrogen peroxide (H_2_O_2_), and hydroxy radical (·OH). These ROS cause the degradation and peroxidation of cell membranes, damaging their structure and function resulting in nucleic acid damage, enzyme inactivation, and ultimately hampering plant growth and development (Miller et al., 2010; Xu et al., 2021). In addition, under normal circumstances, plants regulate ion balance by expelling Na^+^ and absorbing K^+^ to maintain the cytoplasmic Na^+^/K^+^ ratio (Sun et al., 2022b), but the ion balance is destroyed under K^+^ deficiency stress and results in a high Na^+^/K^+^ ratio, which disrupts enzymatic functions that are normally activated by K^+^ in plant cells (Munns et al., 2006).

Many physiological and molecular mechanisms have been reported in plants to cope with K^+^ deficiency stress. Firstly, plants modulate cell osmotic potential through the accumulation of osmolytes such as proline, betaine, soluble sugars, polyamines, and soluble proteins (Yang and Guo, 2018). For instance, under K^+^ deficiency stress, oil palm underwent a reconfiguration of sugar export and induced the accumulation of catecholamine in its leaves to adapt to the K^+^-deficient environment (Cui et al., 2020). Similarly, coconut seedlings experiencing potassium deficiency exhibited elevated levels of phenolic acids, nucleic acids, sugars, and alkaloid-related metabolites (Lu et al., 2023). Plants have also developed both enzymatic and non-enzymatic systems to counteract oxidative damage by eliminating ROS (Ahmad et al., 2010). The enzymatic system primarily consists of superoxide dismutase (SOD), peroxidase (POD), and catalase (CAT), while the non-enzymatic system mainly includes ascorbic acid, alkaloids, carotenoids, and flavonoids (Tan et al., 2006; Ahmad et al., 2010). For example, under K^+^ deficiency stress, maize enhances the activity of antioxidant enzymes to regulate ROS homeostasis and mitigate oxidative damage to photosynthetic organs, thereby sustaining normal photosynthesis (Du et al., 2019). Studies had shown that the transmembrane K^+^ transport rate significantly affects nutrient efficiency, determined directly by the structure and function of K^+^ channels (Wang et al., 2013). It is essential to regulate the synthesis of K^+^ channels or alter their specificity and function to improve K^+^ absorption efficiency under low K stress. Luo et al. (2024) pointed out that overexpression of the high affinity potassium transporter gene *MeHAK5* could improve *Arabidopsis* tolerance to low K^+^ stress. Additionally, when plants cannot acquire additional K^+^, they activate Ca^2+^ signaling pathways, stimulating the activation of K^+^ transporters and channel proteins, ultimately enhancing K^+^ absorption and transport (Hafsi et al., 2017). For example, the cytoplasmic Ca^2+^ concentration in *Arabidopsis* increases under K^+^ deficiency conditions (Behera et al., 2017). The Ca^2+^-dependent CBL1/9-CIPK23 complex facilitates the uptake of K^+^ from the environment by activating the plasma membrane K^+^ channel AKT1 and the K^+^ transporter HAK5 (Ragel et al., 2015; Scherzer et al., 2015).

Studies had demonstrated that exogenous substances can effectively mitigate abiotic stress (Su et al., 2020). For instance, the exogenous application of melatonin (MT) has been shown to enhance apple tolerance to KCl and AlCl_3_ stress (Sun et al., 2023; Wang et al., 2023), while brassinosteroid (BR) and strigolactones (SL) can mitigate the damage to apples caused by salt and alkali stress (Ma et al., 2022; Sun et al., 2022b). Similarly, exogenous hormones can alleviate the adverse effects K^+^ deficiency stress on plant growth and physiology. Liu et al. (2019) indicated that exogenous hormones, including IAA, GA, and ABA, could be used as potential tools to improve the K^+^ deficiency tolerance of sweet potato. Resveratrol (Res) is a polyphenolic organic compound with antitoxic properties produced by numerous plants in response to stimulation (Tian and Liu, 2020; Valletta et al., 2021). As a direct antioxidant agent, Res scavenges diverse ROS and secondary organic radicals to protect cellular biomolecules from oxidative damage caused by abiotic stress and biotic stress (Truong et al., 2018). Zheng et al. (2021a) found that Res could strengthen the activities of antioxidant enzymes and eliminate ROS production induced by iron deficiency stress. Additionally, the exogenous application of Res has the potential to improve the salt stress tolerance of citrus seedlings (Kostopoulou et al., 2014). However, it remains uncertain whether Res can mitigate K^+^ deficiency stress in apple plants.

In this study, we investigated the effects of different concentrations of exogenous Res on *M. hupehensis* seedlings under K^+^ deficiency stress. We analyzed physiological aspects, including the photosynthetic system, oxidative damage, osmotic balance, and ion homeostasis, as well as the expression of K^+^ related transporter genes and kinase genes under Res treatment to evaluate whether Res could enhance the tolerance of apple seedlings to K^+^ deficiency stress. Our findings indicated that the exogenous application Res could alleviate K^+^ deficiency stress in apple seedlings by regulating K^+^/Na^+^ homeostasis, osmotic adjustment, and ROS scavenging. This study offers a novel approach to addressing K^+^ deficiency stress and establishes a theoretical foundation for analyzing the mechanism of Res in apples under abiotic stress.

## 2 Materials and methods

### 2.1 Plant materials and growth conditions

Tissue culture seedlings of *M. hupehensis* were cultured on Murashige and Skoog (MS) medium containing 0.5 mg/L indole-3-butyric acid (IBA) and 0.5 mg/L 6-benzylaminopurine (6-BA). The plants were sub-cultured every 3 weeks for a period of 60 d to promote rooting, and then transplanted into soil as described by Zheng et al. (2018). The *M. hupehensis* plants were maintained at 23 ± 2°C under a 16-h light/8-h dark cycle with a light intensity of 150 μmol m^−2^ s^−1^. After 14 days, seedlings exhibiting similar growth status were selected for subsequent treatment involving K^+^ deficiency and exogenous Res.

### 2.2 K deficiency and exogenous Res treatment

A total of 240 *M. hupehensis* plants were evenly divided into six groups. Group I was irrigated with Hoagland nutrient solution (40 μM K^+^) and served as the control. Group II was irrigated with K^+^ deficiency nutrient solution (4 μM K^+^). Groups III to VI received the same treatment as Group II, but with the addition of exogenous Res (Sangon, Shanghai, China) at concentrations of 10, 50, 100, or 500 μM, respectively. Res was dissolved in ethanol to a final concentration of 10 mM and stored at -20°C. The plants were sprayed and irrigated with Res every 2 days. After 33 days of treatment, the phenotype of the apple seedlings was photographed, and measurements were taken for chlorosis rate, plant height, fresh weight, and dry weight. Each experiment was repeated three times for both technical and biological replicates.

### 2.3 Measurement of chlorosis rate, plant height, fresh weight, and dry weight

After 33 days of treatment, the chlorosis rate was determined by calculating the number of etiolated or dead plants as a percentage of the total number of seedlings in each group. Ten seedlings were randomly selected from each group to measure plant height (the distance from the base of the seedling stem to the top bud growth point), fresh weight (seedlings were washed with distilled water, dried with filter paper, and weighed), and dry weight (seedlings were treated at 100°C for 30 minutes, then placed in a breathable paper bag and dried at 65°C until a constant weight was reached). Each experiment was independently repeated three times.

### 2.4 Measurement of chlorophyll content and photosynthetic parameters

After 33 days of K deficiency and exogenous Res treatment, 20 apple plants from each group were randomly selected to determine the chlorophyll content and basic photosynthetic parameters. Four leaves from each seedling were measured. The chlorophyll content was measured using a SPAD-502 Plus (Konica Minolta, Tokyo, Japan) under light conditions. Photosynthesis rate, transpiration rate, and stomatal conductance were measured using the CIRAS-3 portable photosynthetic system (PP Systems, Amesbury, United States). The light intensity was set to 800 μmol·m^-2^·s^-1^, with 50% humidity and temperature of 23°C. Each experiment was independently repeated three times.

### 2.5 Determination of root morphology and index

After 33 days of K^+^ deficiency and exogenous Res treatment, 10 apple plants from each group were randomly selected for root scanning. The roots were cleaned and placed in a sample tray containing a small amount of deionized water. The root phenotype, root surface area, root length, root tip number, and root diameter were analyzed and determined using an LA-2400 root scanner (Regenting Trumentsing, United States). Each experiment included three biological replicates.

### 2.6 Measurement of ROS levels, MDA content, and antioxidant enzyme activity

Twenty apple seedlings were randomly selected from each group to detect ROS levels, including O_2_^-^ and H_2_O_2_. O_2_^-^ was detected with Nitroblue tetrazolium reagent (NBT; Sangon, Shanghai, China) and H_2_O_2_ was detected with 3,3-diaminobenzidine reagent (DAB; Sangon, Shanghai, China) in leaves, as described by Zheng et al. (2017). Additionally, roots were immersed in 5-(and 6)-chlorom-ethyl-2′-7′-dichlorodihy- drofluoresc-ein diacetate acetyl ester (CM-H2DCFDA) (Thermo Fisher, MA, USA) solution for 30 min and rinsed with distilled water for another 30 minutes. The fluorescence of roots was observed and recorded with a live plant fluorescence detector (Vilber Bio Imaging, Paris, France). Malondialdehyde (MDA) content and POD, SOD, and CAT activities were measured as described by Sun et al. (2022a). Each experiment was independently repeated three times.

### 2.7 Determination of electrolyte leakage and osmolyte content

After 33 days of K^+^ deficiency and exogenous Res treatment, fresh leaves (0.5 g) from each group were used to detect electrolyte leakage and osmolytes. Electrolyte leakage was measured as described by Ahmad et al. (2016). The osmolytes, including proline, soluble sugar, and soluble protein, were detected using extraction kits (Grace, Suzhou, China) following the manufacturer’s instructions. Each experiment was repeated three times.

### 2.8 Measurement of mineral element contents

Mineral element contents were measured using 5 g apple plant tissue after 33 days of K^+^ deficiency and exogenous Res treatment. The samples were dried for 30 min at 105°C and then baked continuously at 80°C for 72 h. Subsequently, 0.5 g of the dried seedlings was ground into powder and digested with 12 mL of a mixture of HNO_3_ and HClO_4_ (5:1 ratio), and then diluted with deionized water to 25 mL for the detection of mineral elements. The concentrations of K, Na, Ca, phosphorus (P), Mg, iron (Fe), manganese (Mn), copper (Cu), and zinc (Zn) were determined by inductively coupled plasma emission spectrometry (ICP–ES) (PerkinElmer, Waltham, United States) as described by Su et al. (2020). Each experiment was independently repeated three times.

### 2.9 Measurement of root net K^+^ flux

The real-time net K^+^ flux of apple plant roots from each group was measured using non-invasive micro-test techniques (NMT) (Xuyue Company, Beijing, China) as described by Jing et al. (2021). Prior to the test, the root tips were soaked in test solution (10 mM KCl, 0.1mM CaCl_2_, pH6.0) for 8 minutes and the K^+^ flux sensor was calibrated with two concentration correction fluids (1.5 mM KCl, 0.1mM CaCl2, pH6.0; 15 mM KCl, 0.1mM CaCl2, pH6.0). During the measurement, the K^+^ flux sensor was positioned adjacent to the root tip under a microscope to measure the net flux. The K^+^ flux data was recorded using the imFluxes V2.0 software. Each plant was tested three times, with five plants tested per treatment group.

### 2.10 Real-time quantitative PCR detection

Total RNA from each treatment was extracted using the RNA prep pure Plant Plus kit (Tiangen, Beijing, China). The total RNA was then reversely transcribed into single-stranded cDNA using the SPARKscript II RT Plus Kit (SparkJade, Jinan, China). Real-time quantitative PCR assays were performed using the LightCycler QLR 480 II system (Roche, Rotkreuz, Switzerland). Primers used for qPCR are listed in **Supplementary Table S1**. Apple *Actin* (accession number: MDP0000774288) was used as the internal control. Candidate genes were identified through a comprehensive review and comparison of literature related to potassium deficiency stress. The relative expression levels were calculated using the 2^-ΔΔCt^ method (Livak and Schmittgen, 2001). Each experiment was independently repeated three times.

### 2.11 Statistical analysis

All data were analyzed using Microsoft Excel 2019 software, and statistical computations were carried out using SPSS software via One-way ANOVA, followed by Tukey’s honestly significant difference (HSD) test (IBM, Armonk, NY, USA). Differences were considered statistically significant at *P* < 0.05. Bar charts was created using GraphPad Prism 9.5.

## 3 Results

### 3.1 Effects of exogenous Res on the growth of apple plants under K^+^ deficiency stress

**Figure 1:**
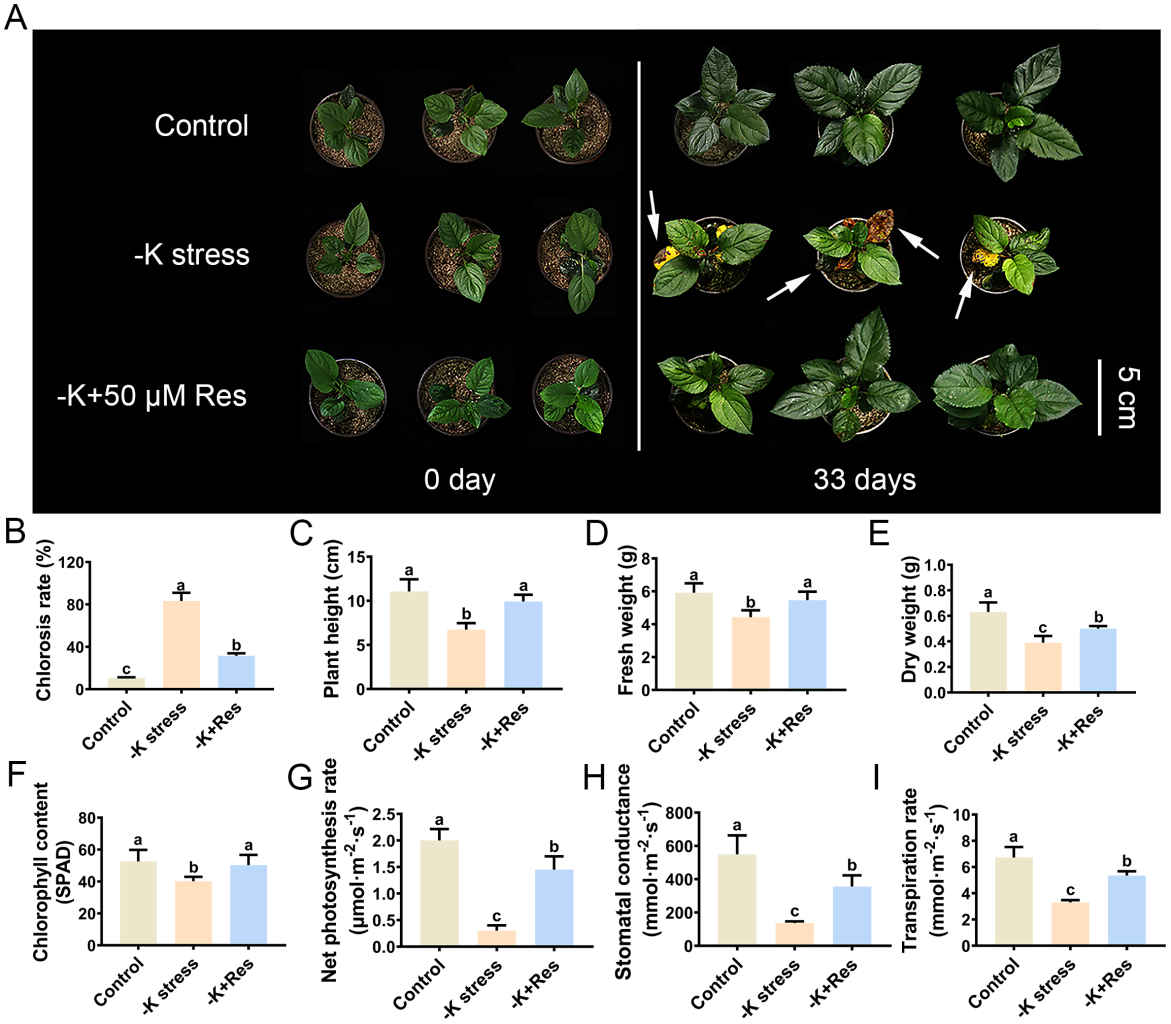
Phenotypic and physiological data of *M. hupehensis* seedlings under k deficiency stress and 50 μM Res treatment. Phenotypes of *M. hupehensis* seedlings treated with K deficiency and exogenous 50 µmol Res on day 0 and day 33 **(A)**, Effect of Res on chlorosis rate **(B)**, plant height **(C)**, fresh weight **(D)**, dry weight **(E)**, chlorophyll content **(F)**, net photosynthesis rate **(G)**, stomatal conductance **(H)**, and transpiration rate **(I)** of apple seedlings after K deficiency stress for 33 days. The bar **(A)** represents 5 cm. The data represent the mean ± SD of biological replicates. Different lowercase letters indicate significant differences according to Fisher’s least significant difference (*P* < 0.05).

In this study, *M. hupehensis* was used to determine the optimal concentration of Res and explore its effects on the growth of apple plants under K^+^ deficiency stress. As shown in **Supplementary Figure S1**, under K^+^ deficiency stress, the leaves of apple seedlings showed high chlorosis rates (86%) and impaired root development. However, this was alleviated when different concentrations of Res were applied (**Supplementary Figures S1A, B**). At low concentrations of Res (10, 50, 100 µM), the chlorosis rates of the apple seedlings significantly decreased from 86% to 55.7%, 37%, and 67.3%, respectively. Conversely, at a high concentration of Res (500 µM), the chlorosis rate was 97.6%, showing no significant difference from the group without Res under K^+^ deficiency stress (**Supplementary Figure S1C**). In addition, the fresh weight of apple plants, which was severely affected by K^+^ deficiency stress, significantly improved with low concentrations of Res, particularly at 50 µM (**Supplementary Figure S1D**). In conclusion, the treatment with 50 µM exogenous Res exhibited the best phenotype and was selected for further research. When transplanted into the soil, the seedlings still exhibited high chlorosis rates under K^+^ deficiency stress. However, these rates significantly decreased from 83.6% to 31.6% after the application of 50 µM Res, with the leaves remaining green (**Figures 1A, B**). Furthermore, plant height, fresh weight, and dry weight were all remarkably affected under K^+^ deficiency stress for 33 days. Plant height decreased from 11.1 cm to 6.7 cm under K^+^ deficiency stress but recovered to 9.9 cm after treatment with 50 µM Res (**Figure 1C**). Fresh weight and dry weight decreased from 5.92 g and 0.63 g to 4.43 g and 0.39 g, respectively, under K^+^ deficiency stress, but were restored to 5.46 g and 0.50 g after treatment with 50 µM Res (**Figures 1D, E**). These results indicate that exogenous Res can protect apple plants from K^+^ deficiency stress.

### 3.2 Effects of exogenous Res on chlorophyll content and photosynthetic parameters under K^+^ deficiency stress

Since exogenous Res could alleviate the chlorosis phenotype of plants under K^+^ deficiency stress, we measured chlorophyll content and photosynthetic parameters. As shown in **Figure 1F**, the chlorophyll content of the apple seedlings sharply decreased from 52.53 SPAD to 40.18 SPAD under K^+^ deficiency stress. However, the application of exogenous Res maintained the chlorophyll content at 50.28 SPAD. Moreover, the photosynthetic parameters, including net photosynthesis rate, stomatal conductance, and transpiration rate, followed a similar trend to chlorophyll content. All values were significantly inhibited under K^+^ deficiency stress but increased with the application of exogenous Res (**Figures 1G-I**), particularly the net photosynthesis rate. Under K^+^ deficiency stress, the net photosynthesis rate decreased significantly from 2 to 0.3 µmol·m^-2^ s^-1^ but recovered to 1.45 µmol·m^-2^ s^-1^ when exogenous Res was applied (**Figure 1G**). Furthermore, K^+^ deficiency stress remarkably decreased stomatal conductance and transpiration rate in apple seedlings by 75.17% and 50.95%, respectively. These values increased by 35.22% and 20.49%, respectively, after treatment with exogenous Res (**Figures 1H, I**). These results indicate that exogenous Res can protect chlorophyll content and the photosynthetic system under K^+^ deficiency stress, thereby maintaining the normal green color of apple leaves.

### 3.3 Effects of exogenous Res on the root phenotype of apple plants under K^+^ deficiency stress

**Figure 2:**
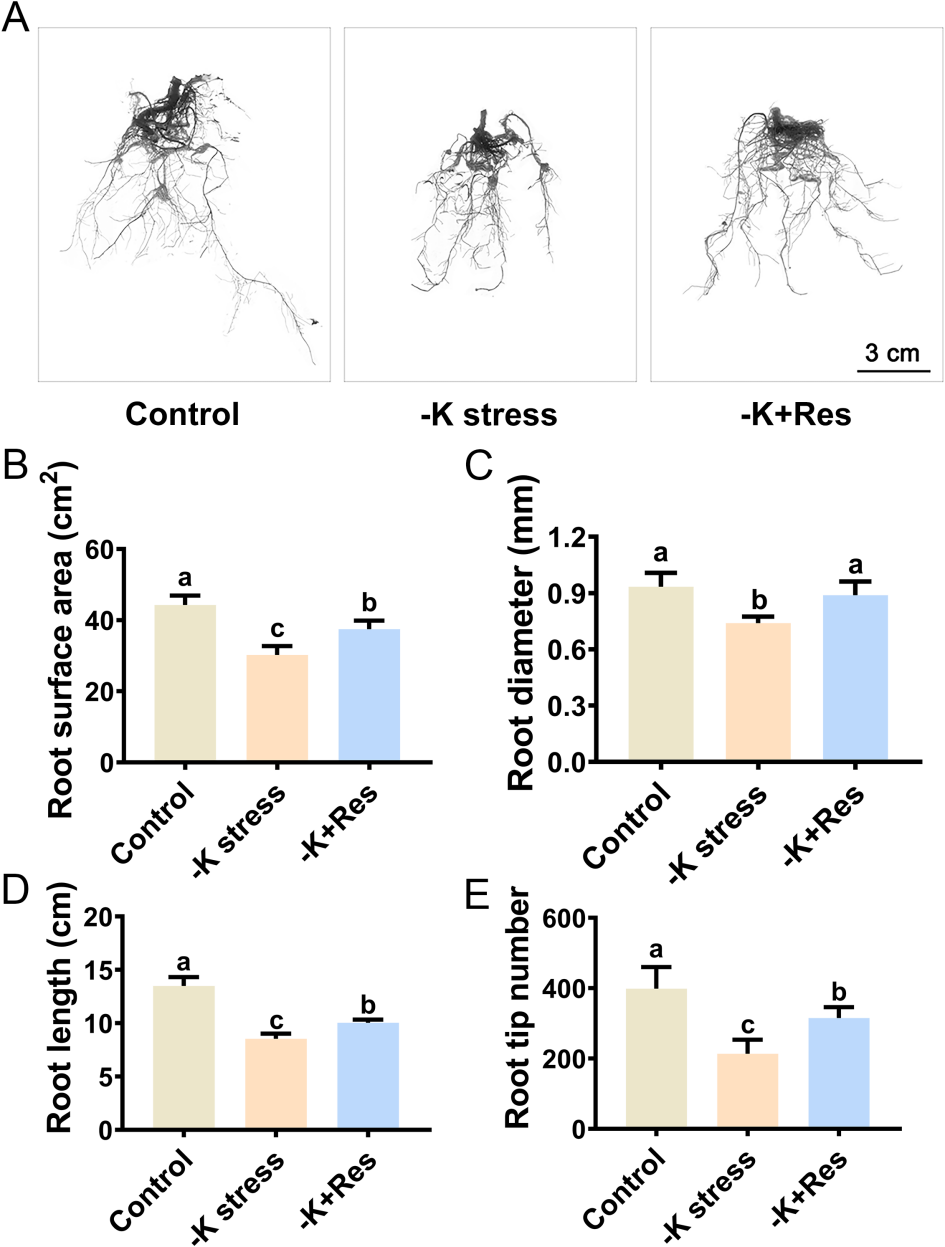
Effects of K deficiency and 50 μM Res on the root growth of acclimated tissue culture seedlings of *M. hupehensis*. The root phenotype **(A)**, root surface area **(B)**, root diameter **(C)**, root length **(D)**, and root tip number **(E)** of *M. hupehensis* acclimated tissue culture seedlings after 33 days of K deficiency stress and Res treatment. The scale is indicated as 3 cm. The data represent the mean ± SD of biological replicates. Different lowercase letters indicate significant differences according to Fisher’s least significant difference (*P* < 0.05).

The root system of the apple plants was examined using a root scanner to investigate the impact of exogenous Res under K^+^ deficiency stress (**Figure 2A**). The root surface area significantly decreased from 44.28 cm^2^ to 30.21 cm^2^ under K^+^ deficiency stress for 33 days, but recovered to 37.49 cm^2^ with the application of exogenous Res (**Figure 2B**). Similarly, root diameter significantly decreased by 17.2% under K^+^ deficiency stress but returned to normal levels when exogenous Res was applied (**Figure 2C**). In addition, the changes in root length and root tip number followed a similar pattern. Root length and root tip number significantly reduced from 13.49 cm and 399 to 8.53 cm and 214 cm, respectively, under K^+^ deficiency stress. The application of exogenous Res partially alleviated these reductions (**Figures 2D, E**).

### 3.4 Effects of exogenous Res on the oxidative damage and antioxidant enzyme activity in apple plants under K^+^ deficiency stress

**Figure 3:**
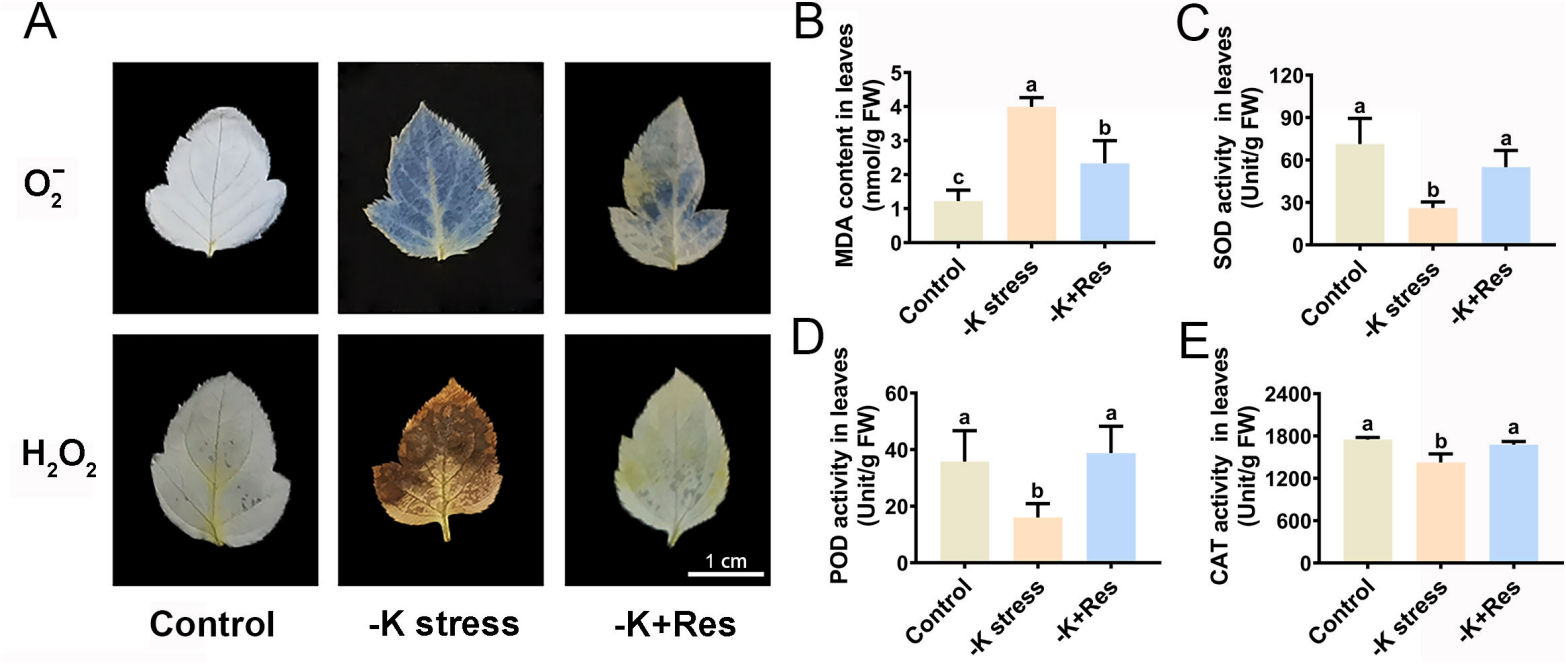
Effects of K deficiency and 50 μM Res on the leaf oxidation index of *M. hupehensis* seedlings. The O_2_^-^ and H_2_O_2_ staining **(A)**, MDA content **(B)**, SOD activities **(C)**, POD activities **(D)** and CAT activities **(E)** in the leaves of *M. hupehensis* after 33 days of K deficiency stress and Res treatment. The scale bar represents 1 cm. The data represent the mean ± SD of biological replicates. Different lowercase letters indicate significant differences according to Fisher’s least significant difference (*P* < 0.05).

K^+^ deficiency stress causes oxidative damage to plants. Therefore, oxidative damage and antioxidant enzyme activities in apple plants were determined after K^+^ deficiency stress and exogenous Res treatment. The staining results of O_2_^-^ and H_2_O_2_ revealed that the leaves of apple plants were seriously damaged by K^+^ deficiency stress. However, exogenous Res significantly reduces the levels of O_2_^-^ and H_2_O_2_ (**Figure 3A**). The trend in MDA content mirrored that of O_2_^-^ and H_2_O_2_ under K^+^ deficiency stress and exogenous Res treatment. The MDA content in the leaves under K^+^ deficiency stress increased from 1.22 to 3.99 nmol/g but decreased to 2.33 nmol/g with the application of exogenous Res (**Figure 3B**). In addition, the activities of antioxidant enzymes (SOD, POD, and CAT) in apple leaves were measured and showed a similar trend (**Figures 3C-E**). SOD, POD, and CAT activities significantly decreased from 71.23, 35.75, and 1749 U/g to 26, 16, and 1426 U/g, respectively, under K^+^ deficiency stress. However, when exogenous Res was applied, the activities of these enzymes were returned to normal levels compared to the control group.

**Figure 4:**
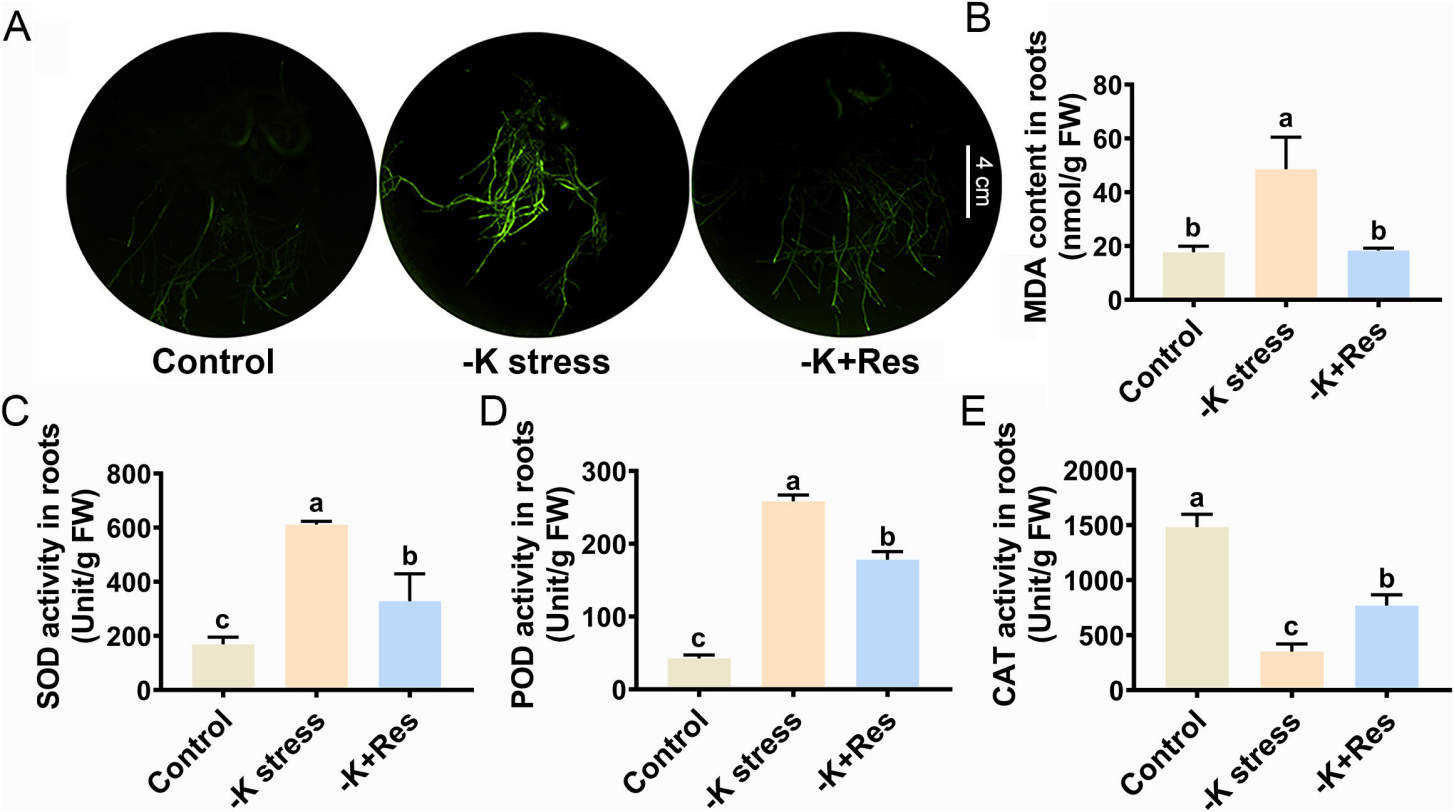
Effects of K deficiency and 50 μM Res on the oxidation index of *M. hupehensis* roots. The O_2_^-^ and H_2_O_2_ staining **(A)**, MDA content **(B)**, SOD activities **(C)**, POD activities **(D)** and CAT activities **(E)** in the roots of *M. hupehensis* after 33 days of K deficiency stress and Res treatment. The scale bar represents 4 cm. The data represent the mean ± SD of biological replicates. Different lowercase letters indicate significant differences according to Fisher’s least significant difference (*P* < 0.05).

ROS staining and antioxidant enzyme activities were also examined in apple roots. The staining results for ROS in roots showed a trend similar to that in leaves, with exogenous Res reducing ROS content in plant roots under K^+^ deficiency stress (**Figure 4A**). The MDA content in roots under K^+^ deficiency stress increased from 17.71 to 48.5 nmol/g but decreased to 18.3 nmol/g under exogenous Res application (**Figure 4B**). The trend of SOD and POD activities in roots differed from those in leaves. SOD and POD activities significantly increased from 168.9 and 42.5 U/g to 611.5 and 258.2 U/g under K^+^ deficiency stress but decreased to 328.5 and 178.1 U/g with the application of exogenous Res (**Figures 4C, D**). However, the trend in CAT activity was similar to that in leaves. CAT activity significantly decreased from 1482.7 to 350.8 U/g under K^+^ deficiency stress but increased twofold compared to the group without Res under K^+^ deficiency stress when exogenous Res was applied (**Figure 4E**). These results indicate that Res enhances the activities of SOD, POD, and CAT in leaves and CAT in roots of apple plants under K^+^ deficiency stress, thereby removing ROS.

### 3.5 Effects of exogenous Res on electrolyte leakage and osmolytes in apple plants under K^+^ deficiency stress

**Figure 5:**
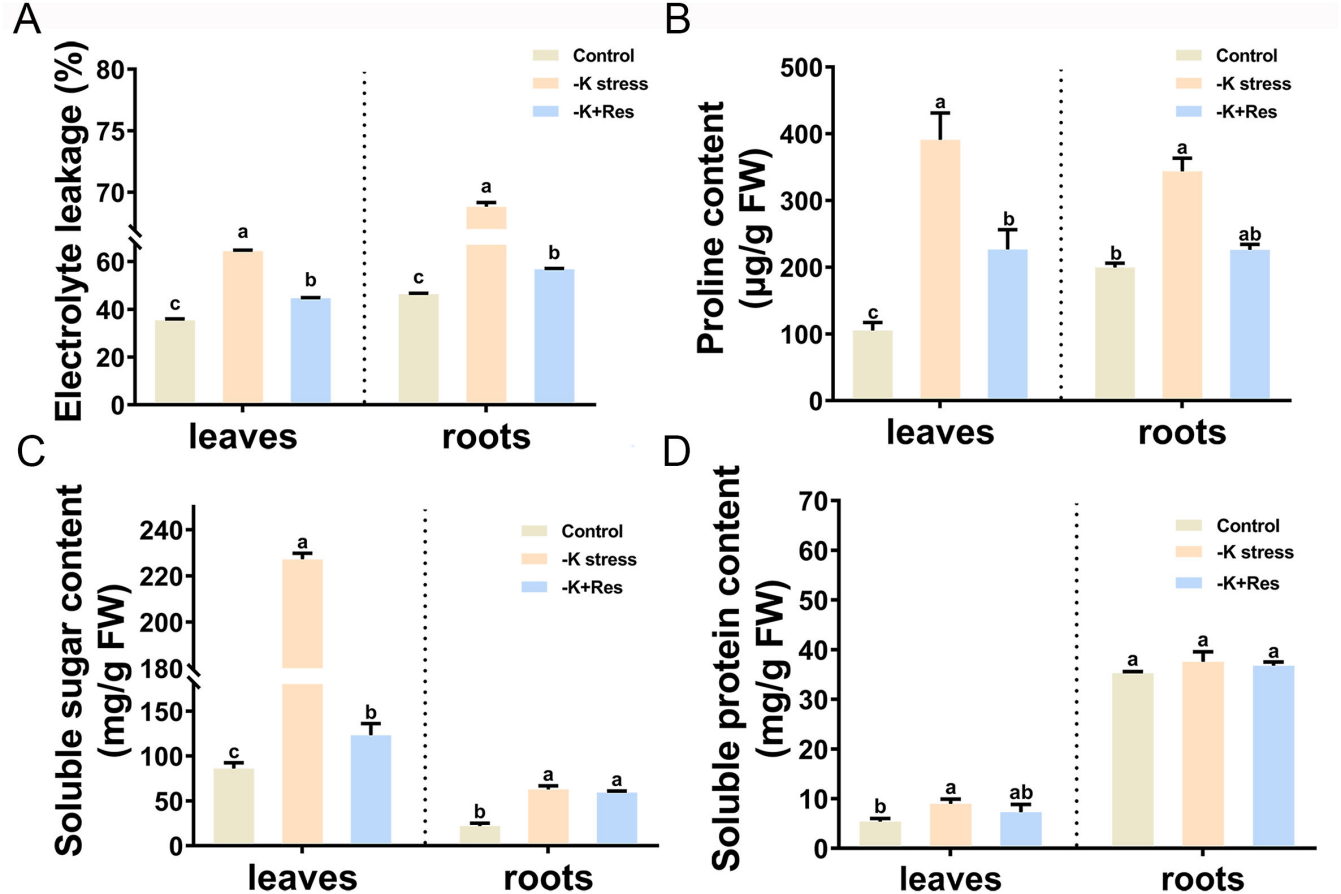
Effects of K deficiency and 50 μM Res on osmotic substances of *M. hupehensis* seedlings. The electrolyte permeability **(A)**, proline content **(B)**, soluble sugar content **(C)**, and soluble protein content **(D)** in the leaves and roots of *M. hupehensis* seedlings after 33 days of K deficiency stress and Res treatment. The data represent the mean ± SD of biological replicates. Different lowercase letters indicate significant differences according to Fisher’s least significant difference (*P* < 0.05).

Electrolyte leakage was measured in the leaves and roots of apple plants treated with K^+^ deficiency and exogenous Res after 33 days. Under K^+^ deficiency stress, leaf electrolyte leakage increased from 35.4% to 64.4%, but decreased to 44.7% after exogenous Res application. Similarly, root electrolyte leakage increased from 46.4% to 68.8%, and then decreased to 56.8% following exogenous Res treatment (**Figure 5A**).

The osmolyte contents in the leaves and roots were also measured under K^+^ deficiency stress and exogenous Res treatment. Proline content in leaves and roots significantly increased from 105.3 and 199.8 µg/g to 390.9 and 343.6 µg/g under K^+^ deficiency stress, but decreased to 226.8 and 225.9 µg/g after exogenous Res treatment (**Figure 5B**). Soluble sugar content in both leaves and roots changed significantly, especially in leaves, which significantly increased from 86.1 to 227.2 mg/g and decreased to 123.2 mg/g under K^+^ deficiency stress, while there was no significant change in roots after exogenous Res treatment (**Figure 5C**). The soluble protein content in leaves increased under K^+^ deficiency stress and returned to normal levels after exogenous Res treatment, whereas root protein content did not change significantly before or after treatment (**Figure 5D**).

### 3.6 Effects of exogenous Res on mineral element contents and K^+^ homeostasis in apple plants under K^+^ deficiency stress

**Figure 6:**
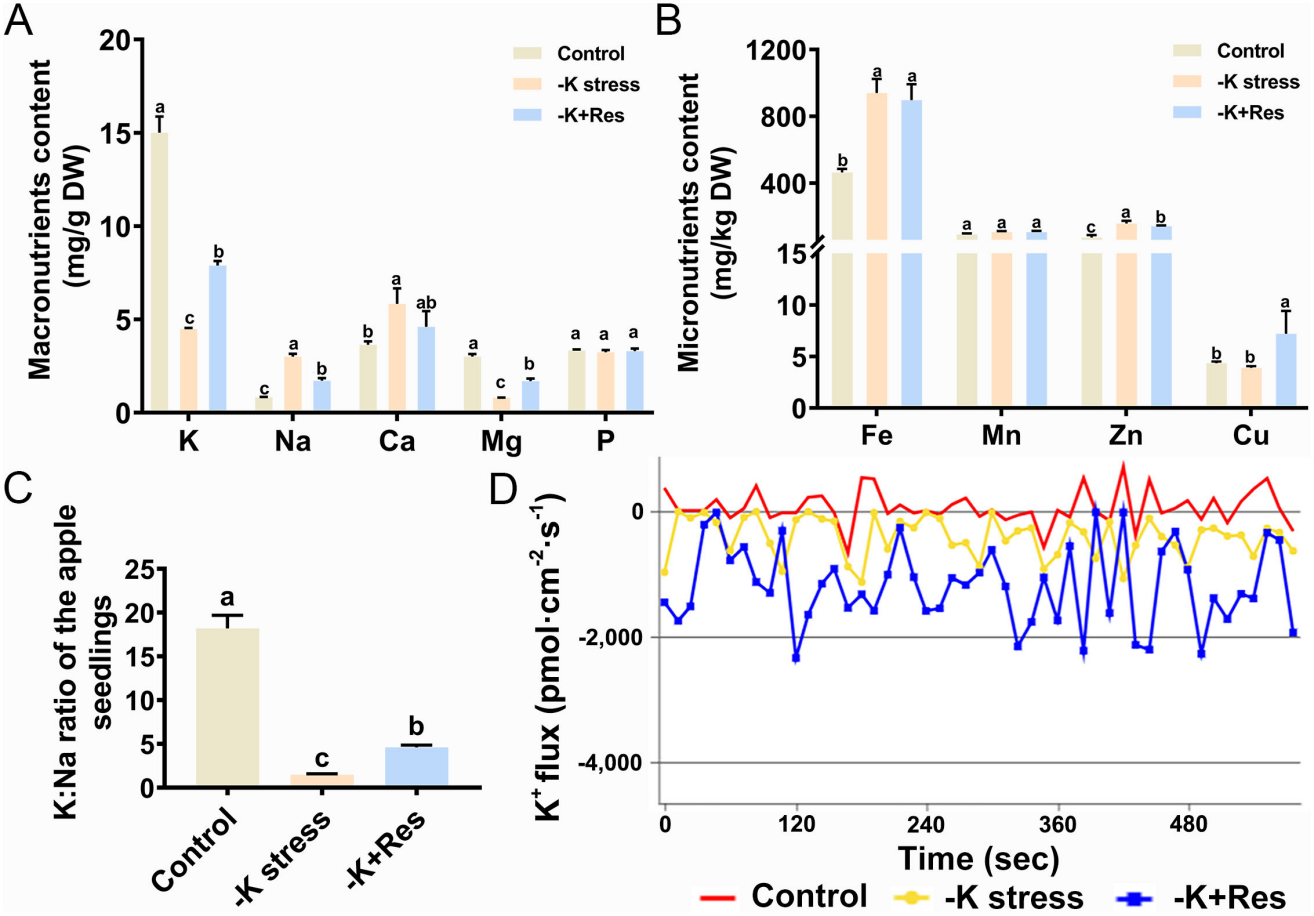
Effects of K deficiency and 50 μM Res on mineral elements content in *M. hupehensis* seedlings. Macronutrients contents **(A)**, Micronutrients contents **(B)**, K:Na ratio **(C)** and K^+^ flux **(D)** of the *M. hupehensis* seedlings after 33 days of K deficiency stress and Res treatment. The data represent the mean ± SD of biological replicates. Different lowercase letters indicate significant differences according to Fisher’s least significant difference (*P* < 0.05).

We measured the macronutrient and micronutrient contents in apple plants under K^+^ deficiency and after exogenous Res treatment. K^+^ content decreased from 15.01 to 4.48 mg/g under K^+^ deficiency stress and increased to 7.89 mg/g after exogenous Res treatment. The trend for Mg content was similar to that of K. Conversely, Na and Ca contents increased from 0.83 and 3.64 mg/g to 3.01 and 5.84 mg/g, respectively, under K^+^ deficiency stress, then decreased to 1.71 and 4.6 mg/g after exogenous Res treatment. There was no significant change in P content (**Figure 6A**). For micronutrients, Fe and Zn contents doubled under K^+^ deficiency stress, but Fe content did not decrease after exogenous Res treatment, while Zn content was significantly decreased. There was no significant change in Mn content under K^+^ deficiency stress, and Cu content increased twofold with exogenous Res application (**Figure 6B**). We also examined the K:Na ratio, an important physiological indicator of plant stress tolerance. Under K^+^ deficiency stress, the K:Na ratio decreased 12.2-fold compared to the control. However, this ratio increased from 1.49 to 4.61 after exogenous Res treatment (**Figure 6C**).

Additionally, we examined K^+^ flux in apple roots under K^+^ deficiency stress and exogenous Res treatment. The positive values of this experiment indicate ion efflux, while the negative values indicate ion influx. As shown in **Figure 6D**, the average of K^+^ flux in apple roots was 73.85 pmol·cm^-2^·s^-1^ under control condition and it turned into influx with a flux of -397.74 pmol·cm^-2^·s^-1^ under K^+^ deficiency stress. When exogenous Res was applied, K^+^ flux decreased to -1178.33 pmol·cm^-2^·s^-1^ compared to K^+^ deficiency stress alone. These results indicate that K^+^ influx increases under K^+^ deficiency stress and that exogenous Res significantly enhances K^+^ influx under these conditions (**Figure 6D**).

### 3.7 Effects of exogenous Res on the expression of related genes in apple plants under K^+^ deficiency stress

**Figure 7:**
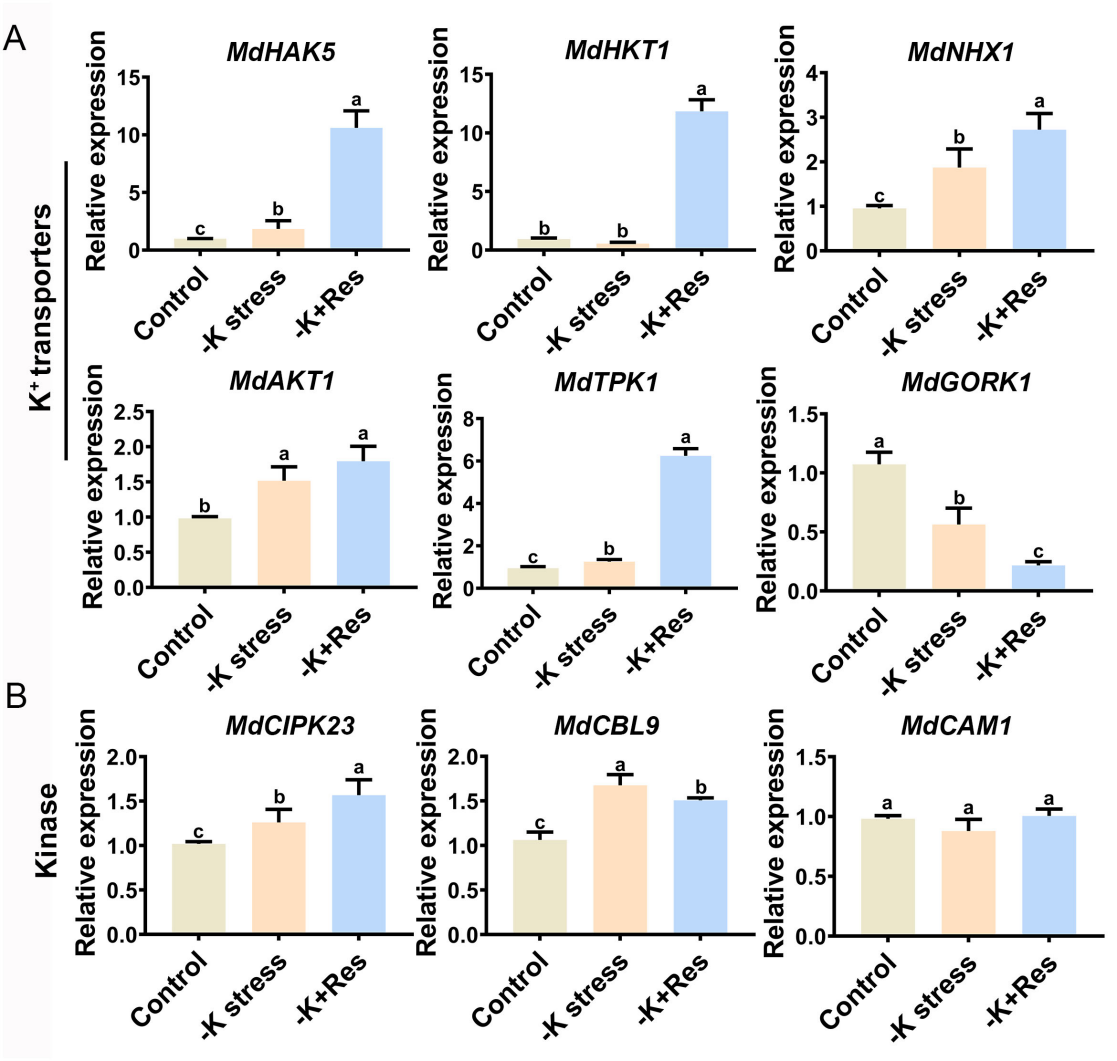
Effects of K deficiency stress and 50 μM Res treatment on gene expression levels in *M. hupehensis* seedlings. Candidate genes were divided into K^+^ transporters genes **(A)** and Ca^2+^ signaling pathway kinase genes **(B)**. The data represent the mean ± SD of biological replicates. Different lowercase letters indicate significant differences according to Fisher’s least significant difference (*P* < 0.05).

As shown in **Figure 7**, we examined the expression levels of nine genes related to K^+^ deficiency response and K^+^ transport in the leaves of *M. hupehensis*, including six K^+^ transporter genes (*MdAKT1*, *MdHAK5*, *MdHKT1*, *MdNHX1*, *MdTPK1*, *MdGORK1*), tow related kinase genes (*MdCIPK23*, *MdCAM1*), and one Ca^2+^-sensor gene (*MdCBL9*). Among these, the expression levels of *MdAKT1*, *MdHAK5*, *MdNHX1*, and *MdTPK1* were significantly up-regulated under K^+^ deficiency stress and further up-regulated after exogenous Res treatment. The expression level of *MdHKT1* showed no change under K^+^ deficiency stress but increased significantly when exogenous Res was applied. Conversely, the expression of *MdGORK1* was significantly down-regulated under K^+^ deficiency stress and further down-regulated after exogenous Res treatment. Regarding the three kinase genes, the expressions of *MdCIPK23* and *MdCBL9* were up-regulated under K^+^ deficiency stress. However, *MdCIPK23* was further up-regulated, while *MdCBL9* was down-regulated after exogenous Res treatment. There was no significant change in the expression of *MdCAM1*. These results indicate that Res influences K^+^ transport and alleviates K^+^ deficiency stress by modulating the expression of specific K^+^ transporter and kinase genes.

## 4 Discussion

Insufficient K^+^ levels in apple plants can lead to inhibited plant growth, inferior fruit quality, and reduced yield (Marschner and Cakmak, 1989). The most direct approach to address K^+^ deficiency involves the application of K fertilizers. However, excessive use of K fertilizer can result in environmental pollution and K salt stress (Zheng et al., 2021b; Sun et al., 2023). Therefore, it is crucial to explore novel solutions for mitigating the K^+^ deficiency stress in apples.

Exogenous substances, such as plant growth regulators, osmotic regulators, and antioxidants, have been found effective in mitigating abiotic stress (Li et al., 2012; Chen et al., 2019b; Hamani et al., 2020). Resveratrol (Res), known as a plant antitoxin, has shown potential in alleviating damage caused by abiotic stress when externally applied (Kostopoulou et al., 2014; Li et al., 2021). However, the effect of Res on K^+^ deficiency stress in plants remains unexplored. Our results demonstrate that the application of Res can alleviate symptoms associated with K^+^ deficiency stress in apple plants by reducing the yellowing rate, enhancing fresh weight, and promoting root growth. Among the different concentrations tested, Res at 50 μM exhibited the most favorable effect on improving the plant growth phenotype. However, when the concentration of Res exceeded 50 μM, the elevated Res concentration further exacerbated the detrimental effects on plant growth, demonstrating a biphasic response (**Supplementary Figure S1**). This suggests that plant growth regulators usually affect plant growth and development in a dose-dependent manner. For instance, the effect of applying 100 μM of Res on Fe deficiency tolerance was much better than applying 10 μM and 200 μM of Res (Zheng et al., 2021a).

The mechanism underlying plant response to K^+^ deficiency stress is intricate. K^+^ deficiency can trigger a surge in ROS, which serve as indicators of the extent of oxidative damage to cells (Mittler et al., 2022). Therefore, the antioxidant system plays an important role in mitigating K^+^ deficiency stress in plants. In this study, based on ROS staining in leaves and roots, it was evident that the levels of O_2_^-^, H_2_O_2_, and MDA significantly increased under K^+^ deficiency stress. Exogenous Res effectively eliminated O_2_^-^ and H_2_O_2_ and reduce MDA content in apple seedlings, suggesting that Res enhances the antioxidant capacity of apple plants and possesses the ability to eliminate ROS. This is consistent with previous studies (Leonard et al., 2003; Soares et al., 2003; Kovacic and Somanathan, 2010; Fernández-Mar et al., 2012). In addition, SOD, POD, and CAT play an important role in the antioxidant processes of plants, and their activity can be enhanced by the application of exogenous substances under stress (Waszczak et al., 2018). For instance, strigolactones have been shown to enhance the activity of antioxidant enzymes under AlCl_3_ stress in apple plants (Zhang et al., 2024). Similarly, in our study, the application of Res enhanced the activity of SOD, POD, and CAT in the leaves of apple plants under K^+^ deficiency stress. Interestingly, there were distinct differences in the activity changes observed between roots and leaves. The activities of SOD and POD were significantly induced by K^+^ deficiency stress, while their activity decreased after Res application in apple roots. We hypothesize this discrepancy might be attributed to the inherent defense mechanisms of plants. As roots are directly exposed to soil, they experience more severe damage compared to leaves. Consequently, plants coordinate a significant amount of antioxidant enzymes to eliminate ROS from roots, resulting in an increase in detected activity levels. Moreover, due to substantial oxidative losses occurring in roots during K^+^ deficiency stress conditions, it is possible that Res application alone may not suffice to counteract these effects effectively, leading to weakened enzyme activity levels. These results suggest that Res may remove ROS by affecting antioxidant enzyme activity; however, when subjected to severe damage caused by K^+^ deficiency stress conditions alone or combined with other factors beyond what can be mitigated by Res application alone, its ability for ROS removal was not enhanced.

Osmotic substances such as proline, soluble sugars, and soluble proteins are effective defense substances against osmotic stress, and their accumulation can relieve osmotic stress in plants (Yu et al., 2024). In this study, the application of Res influenced the levels of proline and soluble sugars in leaves, as well as proline in roots during K^+^ deficiency stress (**Figures 3**, **4**). Furthermore, this study compared the differences in osmotic substance contents between leaves and roots, revealing that the changes in osmotic substances were essentially similar in both tissues. However, under K^+^ deficiency stress, the soluble sugar content in leaves was significantly higher than in roots. This phenomenon could be attributed to insufficient potassium supply leading to the accumulation of soluble sugars in above-ground plant parts, while impeding their transportation to the roots (Pettigrew, 2008).

K^+^ deficiency leads to changes in cytoplasmic Ca^2+^ levels and the transmission of Ca^2+^ signals to Ca^2+^ sensors, such as Ca^2+^-dependent protein kinases (CDPKs), calmodulin (CaM), CaM-like proteins (CMLs), and calcineurin B-like proteins (CBLs), in *Arabidopsis* (Luo et al., 2022). This investigation aimed to assess the expression patterns of specific genes encoding classical calcium signal receptors. CaM plays a crucial role in mediating plant responses to environmental stress through its target protein (Zhou et al., 2016). However, this study indicated that *MdCAM1* did not exhibit significant changes, while *MdCIPK23* and *MdCBL9* were highly expressed after Res application (**Figure 7**). These results suggest that *MdCIKP23* and *MdBCL9* play roles in responding to potassium deficiency stress conditions, while *MdCAM1* did not appear to be significantly involved. In addition, transporting K^+^ plays an important role in this mechanism. The K^+^ transport mechanism in plants is intricate and relies heavily on K^+^ transporters. Therefore, we also assessed the expression levels of certain genes encoding classical K^+^ transporters. *HAK5*, a crucial member of the HAK/KUP/KT family, plays a significant role in response to K^+^ deficiency (Tang et al., 2020). Previous reports have implicated HKT1 and TPK1 in K^+^ absorption (Latz et al., 2013; Jaime-Pérez et al., 2017). In our study, *MdHAK5*, *MdHKT1*, *MdAKT1* and *MdTPK1* were all enhanced by Res application under K^+^ deficiency stress (**Figure 7**). Apart from absorptive transporters for K^+^, there are also excretory transporters such as GORK shaker channel protein family present in plants. In this study, we found that exogenous Res could decrease the expression levels of *MdGORK*. (**Figure 7**). These findings further support the notion that Res enhances cellular capacity for absorbing K^+^, aligning with our determination of the rate of K^+^ flow (**Figure 6D**). Previous studies have shown that K^+^ transporters are regulated by protein kinase CIPKs (Xu et al., 2006; Tang et al., 2020). Based on this, we hypothesized that Res might affect the activity of Ca^2+^-sensor/kinases *MdCBL9* and *MdCIPK23*, thereby influencing the expression of K^+^ transport genes *MdTPK1* and *MdHAK5* to enhance K^+^ uptake, thus alleviating K^+^ deficiency stress.

In summary, the application of an appropriate concentration of Res elicits a positive response in apple plants under potassium deficiency stress. This may be attributed to the potential role of Res in enhancing antioxidant activity, regulating osmotic potential, and modulating K^+^ transporter activity in apple plants.

## Data Availability

The original contributions presented in the study are included in the article/**Supplementary Material**. Further inquiries can be directed to the corresponding author.
