# Supplementary material for: Resveratrol enhances the tolerance of Malus hupehensis to potassium deficiency stress
Source: Front Plant Sci. 2024 Nov 14;15:1503463. doi: 10.3389/fpls.2024.1503463 (PMC11602332; doi:10.3389/fpls.2024.1503463)
Supplement: Supplementary file 1 [file DataSheet1.docx]

***Supplementary material***

1. **Supplementary Figures and Tables**

## Supplementary Figures


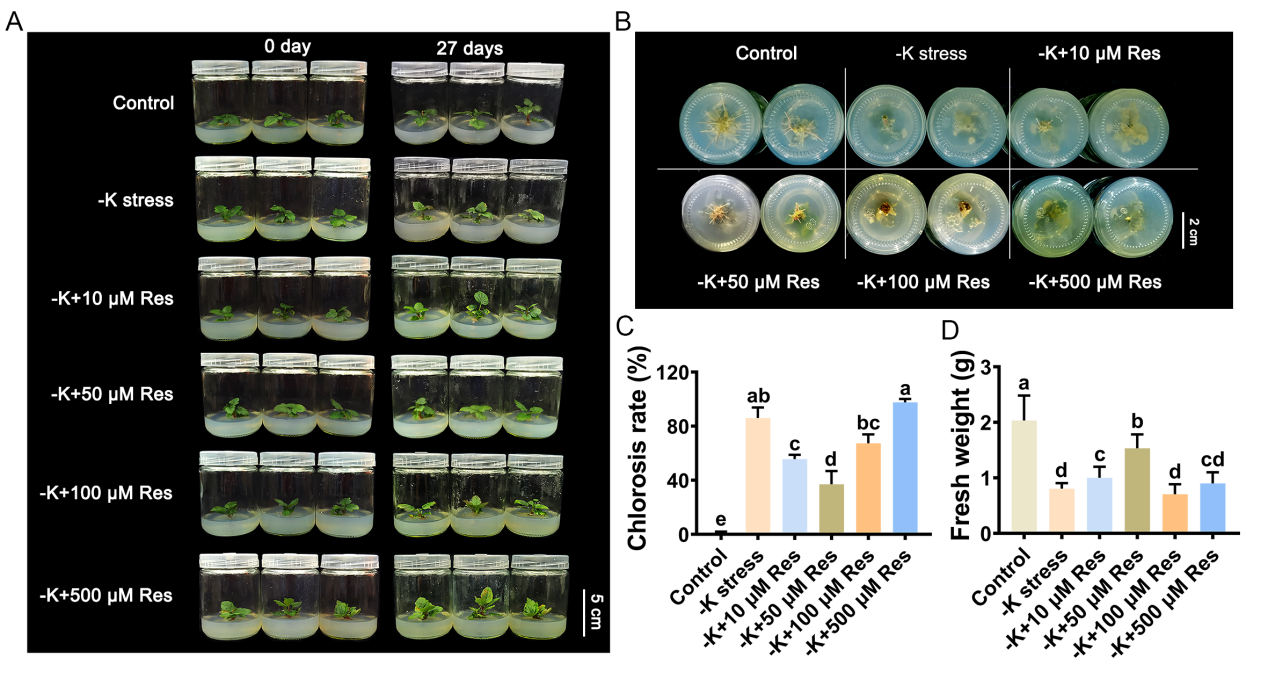


**Supplemental figure S1. The effects of K deficiency and** **different concentrations of Res on the growth of tissue culture seedlings of *M. hupehensis.***

The leaf phenotype (a), root phenotype (b), chlorosis rate (c), and fresh weight (d) of *M. hupehensis* tissue cultur seedlings after 27 days of K deficiency stress and treatment with different concentrations of Res. The bar represents 5 cm (a) and 2 cm (b), respectively. The data represent the mean ± SD of biological replicates. Different lowercase letters indicate significant differences according to Fisher’s least significant difference (*P* < 0.05).

## Supplementary **Tables**

The primers used in this study

**Table S1 Primer sequence**

| Primer name | Forward primer | Reverse primer |
| --- | --- | --- |
| *qMdAKT1* | GAACATGGACAATGCTGGTAGTT | CGACTTTCATCTAGTGGGGTATTTC |
| *qMdHAK5* | ACAGTGATAACACCAACAAAGGC | AATACATACTTGAGGAGCGGGAC |
| *qMdHKT1* | CTTAATCACAAAGACGACAACAGAG | ACATGGAAACTAAAGTAGAACCACC |
| *qMdNHX1* | CTCTGCTGTGGTGAGATTGGG | ACTAAGATGTGTGAGCTTTTGCCTT |
| *qMdTPK1* | TGTCTCATTCCTCCGCCTTCC | CTACGGCGTTATCGTGGGGTAGCAG |
| *qMdGRK1* | TCAAATCKTCGCTCGGGAGCCAATT | GTCCTRCTACATCCAGTATAAAAAG |
| *qMdCIPK23* | ACAAAGGGTATGATGGTGCGAA | CAAGGGGTTAGGATCCAAGATT |
| *qMdCBL9* | GGTGTGATTGACTTCACTGACTTT | TTGTCTATCTTCCCATCTTGATTTA |
| *qMdCAM1* | TTCAAGGAAGCCTTCAGTCTATTC | CTCAGAGTCGGTGTCTTTCATCT |
| *MdActin* | CTTCAATGTGCCTGCCATGTAT | ATTTCCCGTTCAGCAGTAGTG |
